# A case of an unexpected posterior mediastinal functional paraganglioma: case report and literature review

**DOI:** 10.1186/s12871-020-01026-6

**Published:** 2020-05-08

**Authors:** Zhuqing Yang, Qinye Shi, Fangping Bao

**Affiliations:** 1 Department of Anesthesiology, the Fourth Affiliated Hospital of Zhejiang University School of Medicine, N1 Shangcheng Road, Yiwu, Zhejiang Province China; 2 Department of Anethesiology, the First Affiliated Hospital of Zhejiang University School of Medicine, 79 Qingchun Road, Hangzhou, Zhejiang Province China

**Keywords:** Mediastinal tumor, Paraganglioma, Pheochromocytoma

## Abstract

**Background:**

Paraganglioma can be found in a wide range of locations. However, paraganglioma in the posterior mediastinum is rare. An unexpected paraganglioma located in the posterior mediastinum was found during surgery. The anesthesia management of this patient was challenging.

**Case presentation:**

A 65-year-old male with a posterior mediastinal tumor was scheduled for thoracoscopic mediastinal tumor resection. Severe hemodynamic changes during the operation and postoperative pathological diagnosis showed that the patient had a rare case of posterior mediastinal functional paraganglioma, which was not found before the operation. Although the patient did not experience side effects after surgery, he did experience a dangerous surgical process.

**Conclusions:**

The correct diagnosis of paraganglioma, intensive preoperative screening, adequate preoperative preparation, and accurate intraoperative anesthesia management could provide better anesthesia for paraganglioma patients.

## Background

Pheochromocytoma and paraganglioma (PPGL) are a rare class of neuroendocrine tumors that originate from tumors of the adrenal medulla and extra-adrenal sympathetic chain. If the tumor is located in the adrenal gland, it is referred to as pheochromocytoma (PCC), and if it is found outside the gland, it is called paraganglioma (PGL). PCC tumors account for about 80–85% of all PPGLs and synthesize and secrete catecholamines, causing a series of clinical symptoms. PGL accounts for 15–20% of all PPGLs but typically does not produce catecholamines [1, 2]. PGL can be found in a wide range of locations, such as the chest, abdomen, pelvis, and the head and neck. However, PGL is rare in the posterior mediastinum.

In March 2018, a PGL located in the posterior mediastinum was treated in our hospital. This was an unexpected neuroendocrine tumor as we did not diagnose the patient before surgery. Therefore, the patient was operated without receiving adequate preoperative preparation. The patient experienced dramatic fluctuations in hemodynamics during the surgery. The symptoms were treated, and the patient did not experience side effects after surgery. However, he did experience a dangerous surgical process. Therefore, we share this rare disease aiming to inform on how to choose appropriate perioperative management for such patients.

## Case presentation

**Fig. 1:**
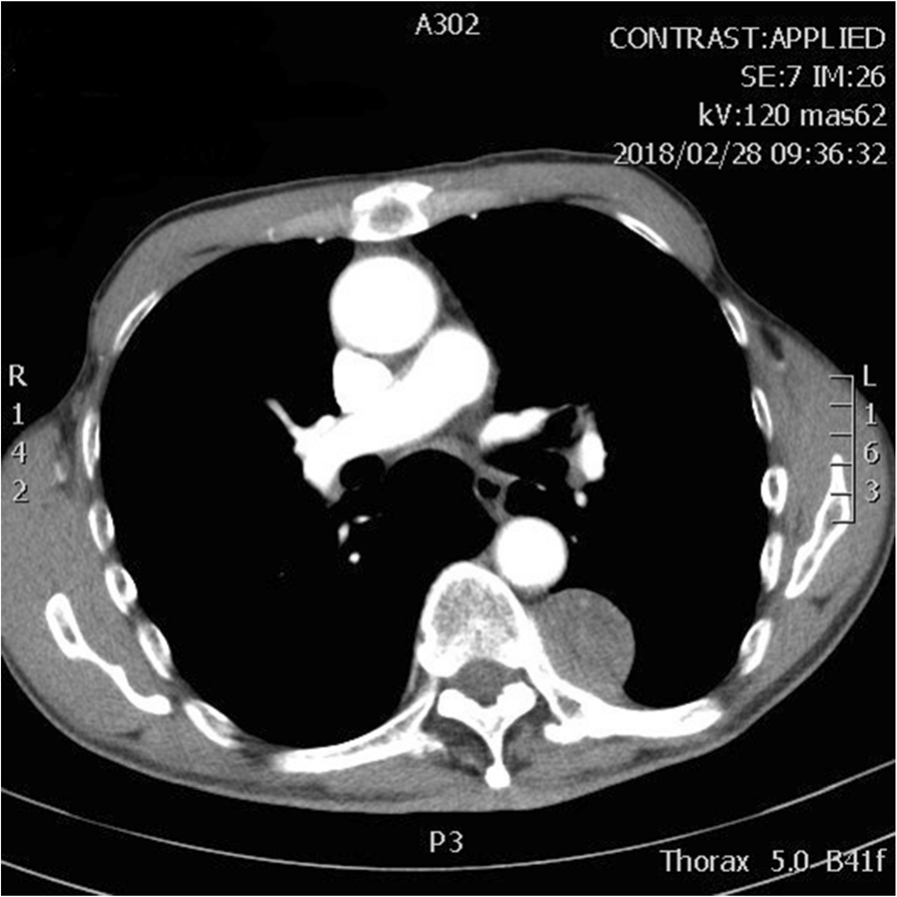
Chest CT. The size of tumor was approximately 4.1 cm × 3.2 cm in the left spinal column, and the enhanced scan showed uniform enhancement

A 65-year-old male was admitted to our hospital for “a mediastinal tumor with cough for more than two months” on March 22, 2018. Two months prior, the patient fell during mountain climbing, resulting in a painful left chest. He was conscious when he was injured and started coughing. He went to the local hospital emergency department, and a chest CT showed a “left posterior mediastinum mass”. The patient received conservative treatment for the pain. As the patient kept experiencing symptoms, such as coughing and feeling uncomfortable, he came to our hospital for further treatment. The patient had a 4-year history of hypertension, for which he took 20 mg/day of sustained-release nifedipine. This was enough to keep his blood pressure stable within the normal range. He had no family history and no history of diabetes, surgery, or smoking and drinking. He is 162 cm in height and weighed 49.3 kg. His physical examination revealed heavy breathing sounds, but no other abnormalities were found. His blood pressure was 138/81 mmHg, heart rate 88 beats/min, respiratory rate 21 beats/min, and temperature 36 °C. His laboratory tests, such as CRP, blood routine, liver and kidney function, tumor markers, and blood gas analysis were all within the normal range. His fasting blood glucose was 7 mmol/l. His chest enhanced CT suggested: 1. Left posterior mediastinal mass, and therefore a neurogenic tumor was considered; 2. Chronic bronchitis, emphysema, multiple pulmonary bullae in the apex of both lungs; 3. Little inflammation in the lower right lung (Fig. 1). No obvious abnormality was found on his ECG, nor by abdominal and cardiac ultrasound. The pulmonary function examination displayed “Mildly restrictive pulmonary ventilation dysfunction”. Given these characteristics, our initial diagnosis was: “left posterior mediastinum tumor; chronic bronchitis; hypertension”. The patient continued to use nifedipine after admission, and his blood pressure was well-controlled.

On March 28, 2018, the patient underwent thoracoscopic mediastinal tumor resection under general anesthesia. When he arrived in the operation room, the invasive arterial blood pressure (ABP), electrocardiogram (ECG), oxygen saturation (SPO_2_), heart rate (HR), and respiratory rate (RR) were measured for baseline values and were continued to be monitored throughout the surgery. The baseline ABP was 147/78 mmHg, HR was 85 beats per min (bpm), and the SPO_2_ was 98%. Intravenous anesthesia was induced and he was intubated successfully. During the operation, intravenous anesthesia, combined with inhalation anesthesia, was used. The patient was placed in the right lateral position for thoracoscopic surgery. The ABP (115–140/64-80 mmHg) and HR (80-89 bpm) were stable but suddenly increased during the exploration of the tumor. His ABP sharply increased to 214/81 mmHg, HR increased to 110 bpm, and the SPO_2_ was steady at 97%. Deeper anesthesia and urapidil (10 mg) iv were used to attempt to control the hypertension, but this was ineffective. The anesthesiologist suspected that the tumor was secreting catecholamines. Nicardipine was given as a continuous infusion at 2 ~ 10 μg/(kg.min) to control the blood pressure. Esmolol 0.5 mg/kg was repeatedly administered to control the heart rate, and 500 ml colloid solution was given to expand the blood volume. When the patient’s blood pressure returned to 140/63 mmHg, and the heart rate dropped to 95 bpm, Nicardipine and Esmolol were discontinued while the tumor was completely removed. After tumor resection, the patient showed circulatory fluctuations again, with the ABP decreasing between 101 and 110/59–80 mmHg and the HR between 95 and 104 bpm. After reducing the depth of the anesthesia, the patient’s blood pressure still showed a decreasing trend (ABP 74/45 mmHg, HR 97 bpm, SPO_2_ 96%). The liquid infusion rate was increased, and 0.03 ~ 0.15 μg/(kg.min) norepinephrine was given to maintain the blood pressure at 100–110/60–70 mmHg. During the whole operation (3 h), a total of 2600 ml liquid was injected into the patient, of which 500 ml was colloidal liquid. Surgical bleeding was 300 ml, and the urine volume was 300 ml. Considering the intraoperative hemodynamic fluctuations, the patient was transferred to the ICU for intensive care after surgery. The vasoactive agents were continuously decreased under surveillance. Four hours after the operation, the patient’s hemodynamics returned to a stable state, and the endotracheal tube was successfully removed. The patient was transferred back to the ward on the first day after surgery. The drainage tube was removed on the third day after the operation, and he was discharged after ten days.

**Fig. 2:**
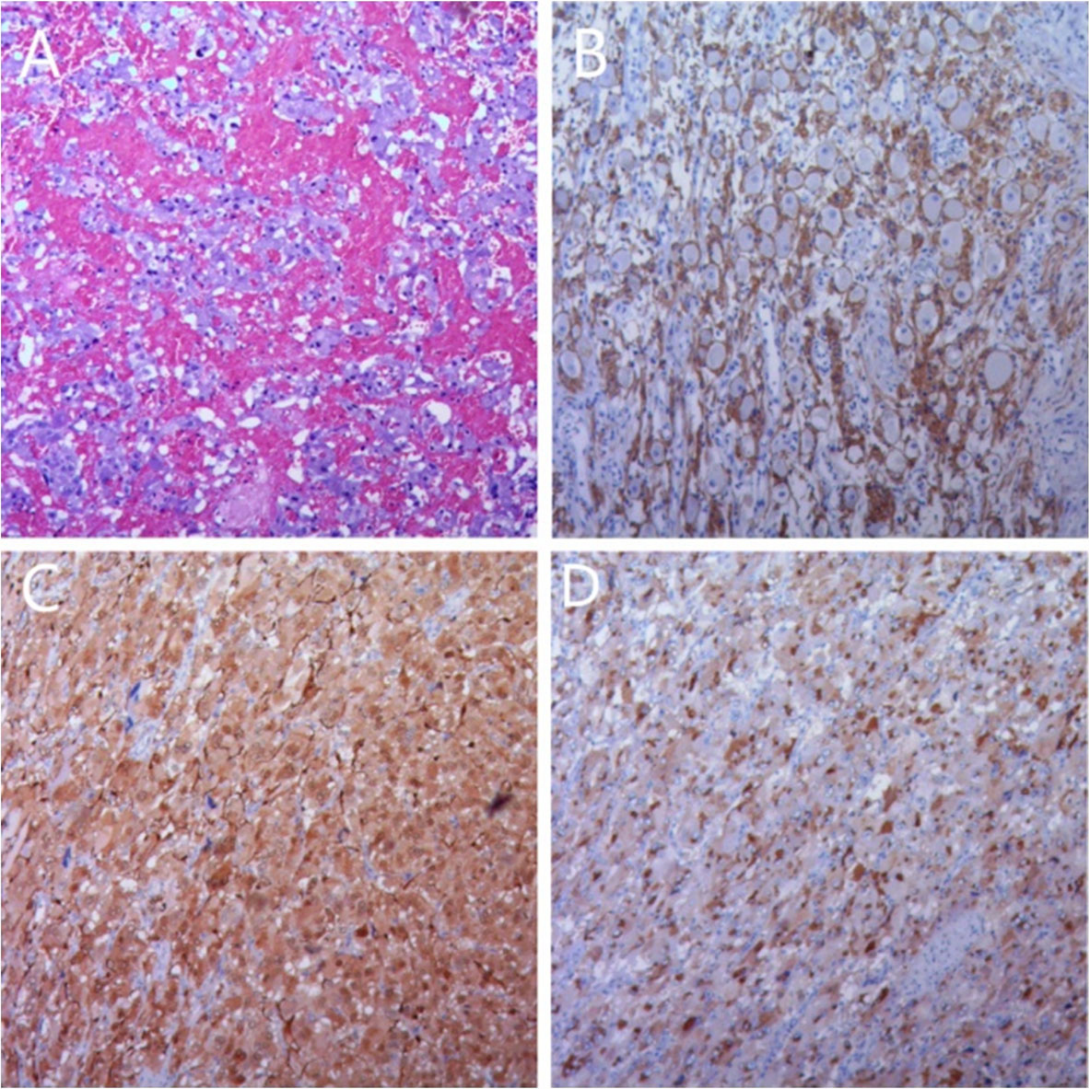
Pathology diagnosed the posterior mediastinum tumor as PGL. **a** Postoperative pathology showed a patchy distribution of tumor cells with blue-purple cytoplasm and abundant interstitial blood sinuses. **b** CD56(+); **c** CgA(+); **d** SYN(+)

Postoperative immunohistochemistry results showed that the tumor was positive for CgA, CD56, NSE, and SYN (Fig. 2), and negative for CK (AE1/AE3), MelanA, ki-67, and s-100. The mass was diagnosed as PGL by postoperative immunohistochemical tests (neuroendocrine markers (CgA, CD56, SYN) were positive) and by a chest enhanced CT scan (uniform enhancement of the tumor).

The patient was followed up in the outpatient clinic for over a year and did not present with recurrences or metastasis of the tumor, and his blood pressure and heart rate remained stable at 135/77 mmHg and 76 bpm with o.p. sustained-release nifedipine.

## Discussion and conclusions

In this case report, we describe an unexpected PGL found during surgery when the patient experienced hemodynamic surges. Severe hemodynamic surges during surgery and the postoperative pathological diagnosis showed that the patient had a rare case of posterior mediastinal functional PGL. This was not detected before the operation, resulting in the patient experiencing a dangerous surgical process.

Clinically, PPGL (PCC and PGL) are incidental adrenal tumors. About 5% of adrenal incidentalomas are PPGLs [3–5]. Due to its secretion of catecholamines, PPGL often presents clinical symptoms such as paroxysmal or persistent hypertension, palpitations, headache, sweating, and hyperglycemia [2, 3]. Between 0.2 and 0.6% of hypertension in patients is caused by PPGL [3]. However, some PPGLs are clinically silent, which makes the diagnosis more difficult [2, 6, 7]. PGL is a neuroendocrine tumor derived from the paraganglia of the sympathetic and parasympathetic nervous systems. They are most commonly found in the head, neck, chest, abdomen, and pelvis, while PGL located in the mediastinum are rare. PGL in rare anatomical locations may cause confusion and diagnostic errors [8]. The patient described here had hypertension, which was well-controlled, without headache, sweating, cold extremities, or other symptoms. For this patient, the lack of symptoms and rare PGL location resulted in missing the diagnosis of PGL. Therefore, the patient did not receive sufficient preoperative preparation, which caused the hemodynamic surges during the surgery.

The primary method for the qualitative diagnosis of PPGL is the detection of hormones and their metabolites [3, 7, 9]. Determination of catecholamine metabolites can improve the sensitivity of the PPGL diagnosis. In addition, PGL/PCC have the highest rate of germline susceptibility, which is at almost 40% [10–12]. Thus genetic testing, combined with clinical manifestations, biochemical, and imaging assessments, can help to understand tumor secretion, tumor malignancy, and develop treatment and follow-up plans for PPGL [12, 13]. For this missed diagnosis, tests for hormones and their metabolites or genetics were not performed.

A previously reported on a patient with bilateral asymptomatic adrenal masses, which were diagnosed as nonfunctional tumors based on the negative laboratory tests (hormones and their metabolites) [14]. Without sufficient preoperative preparation, a hypertensive crisis occurred during the right adrenal resection. Her pathology revealed a pheochromocytoma. Our patient was similar to this case, a silent PGL with severe hemodynamic fluctuations during the operation. Preoperative preparation such as α-adrenergic blockade should be considered in all PGL patients, even if the patient is clinically silent.

PPGL patients’ anesthesia management is very challenging. First, preoperative preparation should be adequate, such as the use of αblockers and augmentation of blood volume [15, 16]. Second, hemodynamic monitoring during the surgery is essential. Monitoring ABP, CVP, the cardiac output (CO), systemic vascular resistance (SVR), and stroke volume variation (SVV) (with the Flotrac/Vigileo system) can provide precise guidance to fluid management. With the guidance of hemodynamic monitoring, adequate fluid therapy and appropriate vasoactive medications can reduce cardiopulmonary complications [17]. Third, appropriate and reasonable use of vasoactive drugs to control perioperative blood pressure fluctuations. Hypertension induced by catecholamine secretion can be treated with the following drugs such as sodium nitroprusside, urapidil, nicardipine, phentolamine, esmolol. Administration of volume expanders and antihypotensive drugs noradrenaline, phenylephrine, vasopressin, or dopamine would be critical in treating hypotension after tumor removal [18]. Fourth, attention should be focused on the clinical manifestations and complications associated with a sudden increase or decrease in catecholamine levels after operation [19]. When encountered intraoperatively with undetected catecholamine secreting PPGL, we should immediately perform adequate hemodynamic monitoring, prepare appropriate vasoactive agents, and give an appropriate amount of fluid treatment.

Fortunately, the patient’s hemodynamic fluctuations were treated promptly, and there were no other life-threatening severe complications. Moreover, what we can learn from this case: (1) The clinical manifestations of PGL patients are diverse, of which 50–80% are often asymptomatic [20, 21]. Patients with a potential mediastinal tumor should be identified from PGL, especially tumors near the vertebral column. Early recognition is essential to manage PGL patients properly. (2) Less hemodynamic monitoring of this patient. We only monitored ABP at that time, and CVP could not be performed as the patient was in a lateral position, and the Flotrac/Vigileo system was not available. As considering the cardiopulmonary complications of over fluid therapies, the fluid dilatation might be insufficient for less hemodynamic monitoring during the operation. This may lead to serious hypotension after the removal of the tumor. (3) Short-acting vasoactive drugs are preferred and recommended to control hypertension, such as sodium nitroprusside, urapidil, and phentolamine. In our first attempt, urapidil was ineffective for reducing hypertension. Sodium nitroprusside and phentolamine were not given priority, because we did not prepare them in advance. Nicardipine, which successfully reduced the blood pressure during the removal of the tumor, can reduce brain and kidney damage, but may lead to serious hypotension after tumor removal as it has a long duration of action. Better drug choices might be more conducive to the patient’s blood pressure management. (4) When facing an unexpected PGL with hemodynamic surges, promptly terminating the operation should be considered. If the hemodynamic surges appear at the entrance of the operation room, anesthesia induction time, changing of position, or during the operation but cannot be controlled, it should be communicated to the surgeons to cancel the surgery. In this case, hemodynamic parameters were stable until the separation of the tumor and could be controlled with vasoactive drugs and, therefore, the surgery continued.

Studies have shown that PGL have the potential to become malignant and are not sensitive to chemotherapy and radiation [3, 22]. Therefore, patients with PGL should be followed up for life after surgical treatment [23]. We followed up this patient for over one year after the operation and found no tumor recurrence or metastasis, and will continue to follow up. The patient’s hypertension has remained stable using the same dose of nifedipine. His hypertension did not recover after the PGL tumor excision, which might confirm that the tumor was a silent PGL.

## Conclusions

In this case report, we discuss a rare posterior mediastinal tumor, which was a functional PGL that was missed before surgery. Our experiences should alert all surgeons and anesthesiologists, to pay attention to the differential diagnosis of PGL, strengthen preoperative screening, and improve intraoperative anesthesia management to provide better anesthesia for such patients.

## Data Availability

All data related to this case report are contained within the manuscript.

## Abbreviations

PPGL: Pheochromocytoma and paraganglioma
PGL: Paraganglioma
PCC: Pheochromocytoma;
ABP: Arterial blood pressure
ECG: Electrocardiogram
SPO_2_: Oxygen saturation
HR: Heart rate
RR: Respiratory rate
bpm: Beats per min
ICU: Intensive care unit
CVP: Central venouspressure
CO: Cardiac output
SVR: Systemic vascular resistance
SVV: Stroke volume variation

## References

[1] Gunawardane PTK, Grossman A (2017). Phaeochromocytoma and paraganglioma. Adv Exp Med Biol.

[2] Neumann HPH, Longo DL, Young WF, Eng C (2019). Pheochromocytoma and Paraganglioma. N Engl J Med.

[3] Lenders JW, Duh QY, Eisenhofer G, Gimenez-Roqueplo AP, Grebe SK, Murad MH (2014). Pheochromocytoma and paraganglioma: an endcrine society clinical practice guideline. J Clin Endocrinol Metab.

[4] Bülow B, Ahrén B (2002). Swedish Research Council Study Group of Endocrine Abdominal Tumours Adrenal incidentaloma experience of a standardized diagnostic programme in the Swedish prospective study. J Intern Med.

[5] Kasperlik-Zaluska AA, Roslonowska E, Slowinska-Srzednicka J, OTTO M, Cichocki A, Cwikla J (2006). 1,111 patients with adrenal incidentalomas observed at a single endocrinological center: incidence of chromaffin tumors. Ann N Y Acad Sci.

[6] Motta-Ramirez GA, Remer EM, Herts BR, Gill IS, Hamrahian AH (2005). Comparison of CT findings in symptomatic and incidentally discovered pheochromocytomas. Am J Roentgenol.

[7] Lenders JW, Pacak K, Walther MM, Walther M (2002). Biochemical diagnosis of pheochromocytoma: which test is best?. JAMA..

[8] Asa SL, Ezzat S, Mete O (2018). The diagnosis and clinical significance of Paragangliomas in unusual locations. J Clin Med.

[9] Nerli RB, Musale A, Ghagane SC, Hiremath MB, Dixit NS (2018). Adrenal hemorrhagic pseudocyst-A case report of a rare presentation of pheochromocytoma. Urol Case Rep.

[10] Castro-Vega LJ, Letouzé E, Burnichon N, Buffet A, Disderot P-H, Khalifa E (2015). Multi-omics analysis defines core genomic alterations in pheochromocytomas and paragangliomas. Nat Commun.

[11] Jochmanova I, Pacak K (2018). Genomic landscape of pheochromocytoma and paraganglioma.Trends. Cancer..

[12] Kantorovich V, Pacak K. New insights on the pathogenesis of paraganglioma and pheochromocytoma. F1000Res. 2018:1–7. 10.12688/f1000research.14568.1. 10.12688/f1000research.14568.1 PMC6173107 30345003

[13] Buffet A, Ben Aim L, Leboulleux S, Drui D, Vezzosi D, Libé R (2019). Positive impact of genetic test on the management and outcome of patients with Paraganglioma and/or Pheochromocytoma. J Clin Endocrinol Metab.

[14] El-Doueihi RZ, Salti I, Maroun-Aouad M, El Hajj A (2019). Bilateral biochemically silent pheochromocytoma, not silent after all. Urol Case Rep.

[15] Ohara N, Kaneko M, Yaguchi Y, Ishiguro H, Ishizaki F, Maruyama R (2018). A case of normotensive incidentally discovered adrenal pheochromocytoma. Clin Case Rep.

[16] Lenders JW, Eisenhofer G, Mannelli M, Pacak K (2005). Phaeochromocytoma. Lancet..

[17] Groeben H, Walz MK, Nottebaum BJ, Alesina PF, Greenwald A, Schumann R (2020). International multicentre review of perioperative management and outcome for catecholamine-producing tumours. Br J Surg.

[18] Ramachandran R, Rewar V (2017). Current perioperative management of pheochromocytomas. Indian J Urol.

[19] Mamilla D, Araque K, Brofferio A, Gonzales M, Sullivan J, Nilubol N (2019). Postoperative Management in Patients with Pheochromocytoma and Paraganglioma. Cancers..

[20] Wald O, Shapira OM, Murar A (2010). Paraganglioma of the mediastinum: challenges in diagnosis and surgical management. J Cardiothorac Surg.

[21] Suzawa K, Yamamoto H, Ichimura K, Toyooka S, Miyoshi S (2014). Asymptomatic but functional paraganglioma of the posterior mediastinum. Ann Thorac Surg.

[22] Stenman A, Zedenius J, Juhlin CC (2019). The Value of Histological Algorithms to Predict the Malignancy Potential of Pheochromocytomas and Abdominal Paragangliomas-A Meta-Analysis and Systematic Review of the Literature. Cancers (Basel).

[23] Alrezk R, Suarez A, Tena I, Pacak K (2018). Update of Pheochromocytoma Syndromes: Genetics, Biochemical Evaluation, and Imaging. Front Endocrinol (Lausanne).

